# Characteristics and Outcomes for Recipients of NVX-CoV2373: A Real-World Retrospective Study in Germany

**DOI:** 10.3390/vaccines12040387

**Published:** 2024-04-06

**Authors:** Lucie Kutikova, James T. Brash, Kawitha Helme, Jack Brewster, Milou Brand, Atif Adam, Sarah Seager, Karel Kostev, Jörg Schelling

**Affiliations:** 1 Novavax Europe, 8001 Zurich, Switzerland; kawithav@gmail.com; 2 IQVIA, London W2 1AF, UK; james.brash@iqvia.com (J.T.B.); jack.brewster@iqvia.com (J.B.); milou.brand@iqvia.com (M.B.); atif.adam@iqvia.com (A.A.); sarah.seager@iqvia.com (S.S.); 3 IQVIA, Epidemiology, 60549 Frankfurt am Main, Germany; 4 Department of Medicine IV, Ludwig Maximilian University of Munich University Hospital, LMU Munich, 80336 Munich, Germany

**Keywords:** real-world evidence, COVID-19, NVX-CoV2373, reactogenicity, effectiveness, duration of protection, outcomes, Germany, NVX-CoV2373, practical vaccination

## Abstract

Real-world evidence supports SARS-CoV-2 vaccination strategies during the COVID-19 pandemic. This real-world retrospective study utilized the German Disease Analyzer database to characterize recipients of NVX-CoV2373 and explore vaccination outcomes. Recipients (≥12 years) of NVX-CoV2373 as a primary series or booster in Germany were vaccinated between March and December 2022. Outcomes included demographics and clinical characteristics of recipients, tolerability/reactogenicity-related events within 7 and 14 days post-vaccination, and protection from COVID-19. Overall, there were 597 recipients (mean age ~60 years) of NVX-CoV2373; 81% were vaccinated by a general practitioner, and 68% had a Standing Committee on Vaccination (STIKO) high-risk factor. The most common baseline comorbidities were chronic neurological (36%) and chronic intestinal (21%) diseases. Among recipients with metabolic disease (~11%), 65% had diabetes. Tolerability/reactogenicity-related symptoms were recorded in ~1% of recipients. There were no sick-leave notes associated with NVX-CoV2373. After 10 months (median, 7 months) of follow-up, 95% (95% CI, 93–95) of recipients were estimated to be protected from COVID-19. Outcomes were similar across the primary series, booster, and STIKO populations. Tolerability and COVID-19 protection support the use of NVX-CoV2373 as a primary/booster vaccination for all authorized populations, including high-risk.

## 1. Introduction

COVID-19, the disease caused by severe acute respiratory syndrome coronavirus-2 (SARS-CoV-2), continues to be a global concern as immune-evasive variants emerge [1]. As such, an evaluation of vaccine use and protection from COVID-19 in real-world populations is needed.

NVX-CoV2373 (Nuvaxovid™; Novavax, Inc., Gaithersburg, MD, USA) is a recombinant COVID-19 vaccine expressing the SARS-CoV-2 spike glycoprotein. This protein-based nanoparticle vaccine is co-formulated with a saponin-based adjuvant, Matrix-M™. In clinical trials, NVX-CoV2373 demonstrated efficacy in preventing COVID-19, providing durable protection after primary series vaccinations in people ≥12 years of age [2,3,4,5]. Immunogenicity data after booster doses of NVX-CoV2373 suggested protection against COVID-19 [6,7], supporting the availability of NVX-CoV2373 in Germany (in March 2022) as a primary series and booster to prevent COVID-19 in those ≥12 years of age. Full market authorization was received from the European Medicines Agency in May 2023 for the ancestral strain and for the XBB.1.5 strain in October 2023 [8]. Furthermore, NVX-CoV2373 is recommended by the Standing Committee on Vaccination (STIKO) as an alternative to mRNA vaccines [9,10].

We report findings from the first real-world retrospective observational database study in Europe performed to characterize recipients of NVX-CoV2373 and to explore the impact of NVX-CoV2373 on tolerability-related events and protection from COVID-19.

## 2. Materials and Methods

### 2.1. Study Design and Recipients

This study was a retrospective, observational, non-interventional analysis of data from the IQVIA™ German Disease Analyzer database in the Observational Medical Outcomes Partnership common data model format. This database contains anonymized electronic medical records, including demographics, diagnoses, and prescriptions generated directly from the computer systems of office-based physicians (general practitioners or specialists) throughout Germany [11]. Agreement between database content and German reference data was demonstrated for major chronic diseases in a 2017 study. At that time, the database was associated with almost 2500 practices and over 7 million patients, and it has since grown. The database has been used for several studies on prescription drug use/effectiveness, medication safety, and COVID-19 and vaccinations. It is representative of the German physician population, based on assessments of the German federal state, community size category, age of physician distribution, and specialist groups. The Disease Analyzer database includes information from 1992 through December 2022.

**Figure 1:**
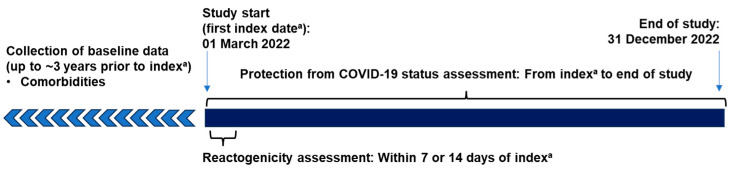
Study design.

Data from individuals ≥12 years of age who received an NVX-CoV2373 primary series/booster in Germany between 1 March 2022 and 31 December 2022 were analyzed; the date of the NVX-CoV2373 vaccination was considered the index date (Figure 1).

Previous recipients of an NVX-CoV2373 full primary series or booster were identified by codes in the database; different codes are listed in the database for first (88335 [A,V]), second (88355 [B,W,H]), and booster (88355 [R,X,K]) doses. Recipients were placed in the primary series group if they had a first or second dose code. It was assumed that if a recipient had a code for a second but not a first dose, they had also received a first dose and were included in the primary series group. The collected data were from multiple office-based physicians/providers across East and West Germany.

### 2.2. Study Objectives

The objectives of this study were to characterize recipients of NVX-CoV2373, explore short-term tolerability/reactogenicity-related events after vaccination, and examine the impact of vaccination with NVX-CoV2373 on protection from COVID-19. Characteristics of recipients included the number receiving NVX-CoV2373 by month of vaccination, length of follow-up from vaccination, and the specialty and location of the provider administering the vaccine. The demographics and clinical characteristics of recipients were also assessed, including age, gender, and comorbidities that define STIKO risk groups for severe COVID-19. Most comorbidities were assessed 3 years prior (including index); psychiatric disorders were assessed 1 year prior (including index); and Down syndrome was assessed any time prior (including index; Table S1). Recipient characteristics were reviewed to determine if they fell into one of the following STIKO high-risk groups: (1) aged ≥60 years; (2) aged ≥18 years with prior diagnosis of one or more comorbidities that increase risk of severe COVID-19; and (3) residents in care facilities, defined as cognitive frailty by dementia, Alzheimer’s disease, or Parkinson’s disease.

### 2.3. Outcomes

Tolerability/reactogenicity-related events were measured within 7 and 14 days of receiving NVX-CoV2373. This included a doctor’s visit for fever, fatigue, malaise, muscle pain, joint pain, nausea/vomiting, or headache, or sick leave from work (sick leave note) linked to symptom diagnosis. Protection from COVID-19 was defined as a lack of doctor’s visit associated with COVID-19 diagnosis from the index date to the end of the study. A COVID-19 diagnosis was determined based on the receipt of a diagnosis code for proven COVID-19 virus detection; the code was based on patient symptoms and/or laboratory-confirmed COVID-19.

### 2.4. Statistical Analysis

Statistical analysis of this study was conducted using Observational Medical Outcomes Partnership analytical tools in structured query language (SQL) via the Snowflake^®^ and OHDSI ATLAS tools (in addition to Snowflake and R). Descriptive statistics were reported using frequency and percentage distributions for categorical variables. Continuous variables were described using mean and median interquartile ranges (IQR) and standard deviation (SD). Results of recipient counts <5 are censored to maintain anonymity. Kaplan–Meier plots were generated to estimate being COVID-19 diagnosis-free after vaccination. Data were stratified by STIKO high-risk groups where applicable and when not limited by sample size (<10 recipients), as predefined in the protocol.

## 3. Results

### 3.1. Recipient Characteristics

Data were extracted for 597 recipients of NVX-CoV2373 as a primary series (58%) or booster (42%). The median follow-up time was 7 months (IQR 3 to 8 months) (Table 1). There was a broad age representation for recipients, with a median age of 58 years (IQR 44–73) for all recipients, 51 years (IQR 39–63) for the primary series group, and 71 years (IQR 56–81) for the booster group. A higher proportion of recipients in the primary series group were female (58%). In the booster group, gender was well-balanced (49% female). Most recipients (81%) were vaccinated by a general practitioner, a proportion consistent with the primary series (81%) and booster (82%) groups. The next most common specialties of physicians administering NVX-CoV2373 were cardiology (4%), gynecology (3%), and pediatrics, pulmonary, neurology, and urology (each 2%). Recipients were distributed throughout Germany (overall population: 56% in East Germany, 44% in West Germany). For the booster group, information on which vaccine was received prior to the index date was available for 87 recipients (35%); the majority (n = 85; 98%) of those who received the prototype monovalent mRNA vaccine BNT162b2.

Overall, 404 recipients (68%) had a STIKO high-risk factor for severe COVID-19. Among all recipients, 46%, 53%, and 3% were in the STIKO ≥ 60 years, STIKO ≥ 18 years + comorbidity, and STIKO care-facility resident groups, respectively (Table 1). A greater proportion of recipients in the booster group (81%) had a STIKO high-risk factor, compared with those in the primary series group (58%).

The demographics and baseline clinical characteristics of recipients were also reviewed by age stratification into three groups: adolescents/young adults aged 12–35 years; adults aged 36–59 years; and older adults aged ≥ 60 years (Table 2). Almost all recipients aged ≥ 60 years (91%) were vaccinated in a general practitioner’s office, compared to 72% and 73% of 12- to 35-year-old and 36- to 59-year-old recipients, respectively; the remainder of vaccinations were administered by a specialist. Approximately half (53%) of recipients under 60 years of age were from West Germany, compared to 33% of recipients aged ≥ 60 years from this region. As expected, the proportion of recipients in the ≥18 years + comorbidity STIKO high-risk group increased with age.

**Figure 2:**
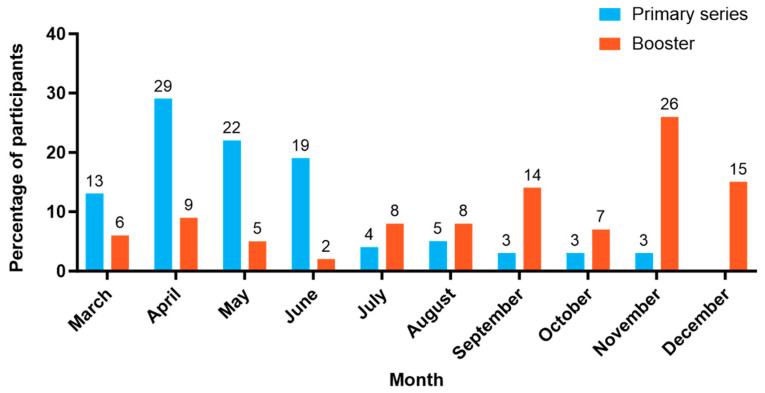
NVX-CoV2373 vaccine uptake by month in 2022.

Of those in the NVX-CoV2373 primary series group, 83% were vaccinated within 4 months of NVX-CoV2373 availability, which could indicate that recipients were waiting for its availability as an alternative COVID-19 vaccine option. For the booster group, 62% were vaccinated in September through December 2022 (Figure 2), which aligns with the expected timing.

Multiple comorbidities were observed in individual recipients (≥1 comorbidity, 53%; ≥2 comorbidity, 30%; ≥3 comorbidity, 17%; Table 3). The most common baseline comorbidities were chronic neurological (36%) and chronic intestinal (21%) diseases. Recipients with chronic respiratory, cardiovascular, or metabolic disease each encompassed 10–11% of the total population; of those with a metabolic disease, the majority (66%) had diabetes. The frequency of several of the comorbidities, including recipients with multiple STIKO high-risk comorbidities, occurred in a higher proportion of recipients in the booster group than those in the primary series group. The most pronounced differences between the primary series group and the booster group were observed with neurological (29% vs. 47%) and cardiovascular (6% vs. 17%) diseases.

**Figure 3:**
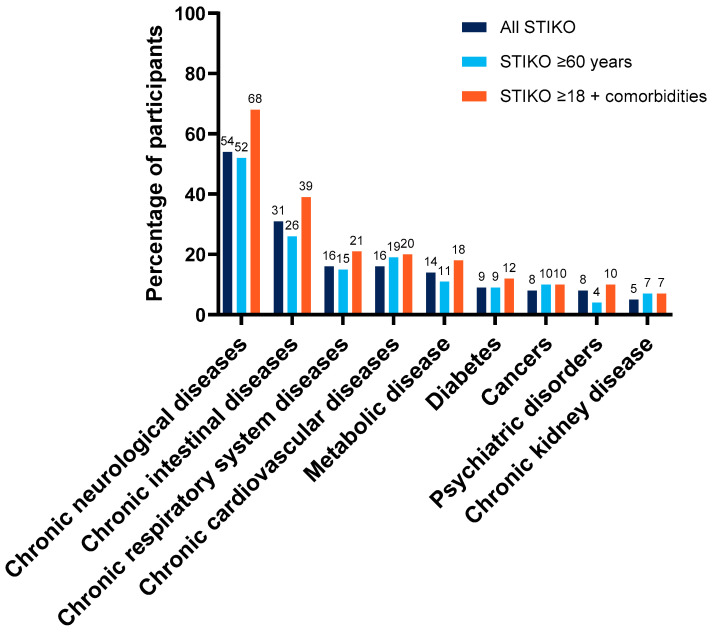
Comorbidities in STIKO populations. Comorbidities occurring in >3% of the overall STIKO population are shown.

As with the total population, chronic neurological disease was the most common in the overall STIKO group (54%), STIKO ≥ 60 years (52%), and STIKO ≥ 18 + comorbidities (68%) groups (Figure 3). Chronic intestinal disease was the next most common comorbidity, occurring in 31%, 26%, and 39% of the overall STIKO, STIKO ≥ 60 years, and STIKO ≥ 18 + comorbidities groups, respectively. Recipients in the STIKO ≥ 18 + comorbidities group had higher or equal frequencies of comorbidities compared to the STIKO ≥ 60 years group. The largest differences between the STIKO ≥ 60 years versus the STIKO ≥ 18 + comorbidities groups were for neurological (52% vs. 68%), intestinal (26% vs. 39%), respiratory (15% vs. 21%), and metabolic (11% vs. 18%) diseases.

### 3.2. Tolerability-Related Outcomes

Among the total population, a similar proportion of tolerability-related doctor’s visits were reported within 7 days (n = 5; <1%) and 14 days (n = 6; 1%) of vaccination. No recipients received a sick-leave note for work related to tolerability. Given the rarity of the events, subgroup analyses were not conducted.

### 3.3. Protection from COVID-19

**Figure 4:**
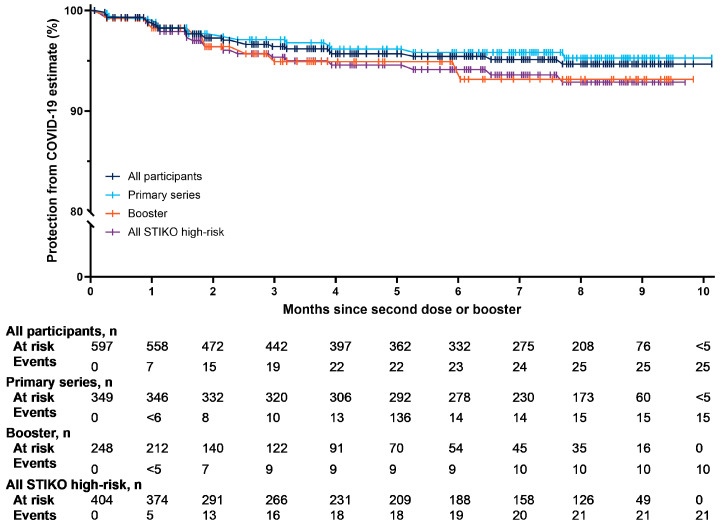
Protection from COVID-19 over time.

After a maximum follow-up of 10 months (median 7 months) after vaccination with NVX-CoV2373, protection from COVID-19 in the total population was estimated at 95% (95% CI, 93–97) (Figure 4). Protection from COVID-19 was similar among recipients of NVX-CoV2373 as part of the primary series (96% [95% CI, 0.94–0.98]) or a booster (93% [95% CI, 0.88–0.98]), and in those with a STIKO high-risk factor (94% [95% CI, 0.91–0.95]).

## 4. Discussion

This retrospective real-world study aimed to characterize recipients of NVX-CoV2373, determine vaccine tolerability, and assess protection from COVID-19. At the time of this report, this is one of the first studies to describe the routine clinical practice use and performance of NVX-CoV2373 in Europe.

With a maximum follow-up of 10 months, protection from COVID-19 was estimated to be 95%; this robust protection confirms strong clinical trial results [3,4,5]. The Disease Analyzer database comprises data from contacts to the healthcare system and/or drug prescriptions; however, most of the COVID-19 cases caused by Omicron variants were mild and did not lead to contact with a general practitioner. Therefore, although high rates of protection were reported from this dataset, this protection rate might be higher than expected.

**Figure 5:**
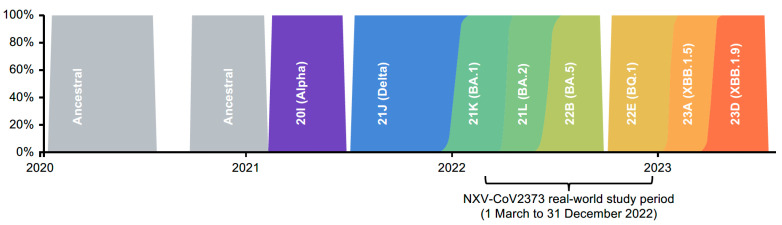
SARS-CoV-2 strain circulation in Germany over time. Figure reproduced with permission from Nextstrain.org. Available at https://nextstrain.org/ncov/gisaid/global/all-time?f_country=Germany (accessed on 4 October 2023).

During this study, the major circulating strains were Omicron subvariants (Figure 5), a key point when considering the waning immunity to emerging variants [12]. This study was conducted while the Alpha, Delta, and early Omicron variants were in circulation and demonstrates the effectiveness of NVX-CoV2373 in a real-world population. The immune composition of the global population related to SARS-CoV-2 is continually evolving based on exposure to new variants/subvariants as well as the receipt of booster doses and updated vaccines. Real-world analyses such as this are informative during these times of dynamic evolution of SARS-CoV-2. Importantly, these analyses should be revisited as recommendations are released for vaccination against the most current circulating variants of interest/concern.

NVX-CoV2373 was widely utilized across different age groups and regardless of comorbidities, the most relevant to COVID-19 being cardiovascular and metabolic conditions. The decision for a general practitioner to recommend a vaccine is dependent on all of the conditions a patient is experiencing; therefore, evidence supporting use in those with comorbidities is critical for decision making. These results support NVX-CoV2373 as a viable option for broader vaccine choice across populations.

At its introduction, there was a larger proportion of recipients of NVX-CoV2373 as a primary series, compared to a booster. This suggests the possibility that recipients were waiting to be vaccinated as they were hesitant to receive an mRNA option [13,14]. Additionally, more booster doses were administered from September 2022 through December 2022, aligning with expectations for booster vaccinations in the fall and winter months. Overall, results were similar for recipients of NVX-CoV2373 as a booster or as a primary series, supporting use of NVX-CoV2373 in either immunization format.

Negligible tolerability-related events were observed at both 7 and 14 days post vaccination with NVX-CoV2373. No sick-leave notes were provided based on symptom diagnosis, which supports a positive economic impact by reducing workplace absenteeism due to COVID-19 [15,16].

Given the retrospective nature of this study, not all relevant information might have been recorded at the time. Due to the nature of the data and the nonrandomized study population, the results might have been affected by selection bias. Prior to 2022, PCR tests were performed outside of the normal healthcare system, in “test centers”. Because these data were not captured in the Disease Analyzer database, infection rates in the study population prior to vaccinations could not be reported. Additionally, data were collected in an outpatient setting, which might impact the interpretation of protection. In this setting, the analyses were not able to capture study outcome data for recipients who were hospitalized or without a doctor’s visit (e.g., visited a testing center), which could result in an overestimation of protection from COVID-19 or an underestimation of tolerability-related symptoms. Testing centers in Germany were providing free services until July 2022, after which the population would have been more inclined to see a physician for testing; however, most cases from variant strains were mild and did not lead to doctors’ visits. Disease Analyzer is a database representative of Germany’s population and pharmaceutical product use [11]; however, low uptake of NVX-CoV2373 prevented analysis of a larger sample size. The relatively small sample size limits the generalizability of the results as well as the ability to conduct a full set of subgroup analyses. Further research with larger sample sizes is warranted.

## 5. Conclusions and Future Directions

These results suggest that individuals receiving NVX-CoV2373 are generally well-distributed and include those with STIKO risk factors for severe COVID-19. A high number of individuals were protected against COVID-19 for up to at least 10 months, with limited tolerability-related events after vaccination. The results from this study align with STIKO recommendations and support the use of NVX-CoV2373 for all authorized populations, including those with comorbidities. The outcomes from this study support NVX-CoV2373 as a viable alternative to mRNA vaccines for both primary series and boosters, broadening vaccine choice.

## Figures and Tables

**Table 1:** Baseline characteristics of NVX-CoV2373 recipients.

Characteristic	All Recipients (n = 597)	Primary Series (n = 349)	Booster (n = 248)
Age, median years (IQR)	58 (44–73)	51 (39–63)	71 (56–81)
Gender, %			
Male	46	42	51
Female	54	58	49
Unknown	NA	NA	0
Median follow-up, months (IQR)	7 (3–8)	8 (7–9)	3 (2–6)
Treating physician, %			
General practitioner	81	81	82
Specialist	19	19	18
Geographical region, %			
East Germany	56	39	81
West Germany	44	61	19
STIKO high-risk group, %	68	58	81
Age ≥ 60 years	46	29	69
Age ≥ 18 years + comorbidity	53	45	65
Care-facility resident	3	NA	NA

COVID-19, coronavirus disease 2019; IQR, interquartile range; NA, not available; STIKO, Standing Committee on Vaccination.

**Table 2:** Baseline characteristics of NVX-CoV2373 recipients, stratified by age groups.

Characteristic	Age 12–35 (n = 78)	Age 36–59 (n = 245)	Age ≥ 60 (n = 274)
Age, median (IQR) years	28 (23–33)	49 (43–56)	74 (67–82)
Gender, %			
Female	53	58	51
Male	47	42	49
Unknown	0	0	NA
Median follow-up, months (IQR)	8 (6–9)	7 (6–8)	4 (2–8)
Treating physician, %			
General practitioner	72	73	91
Specialist	28	27	9
Geographical region, %			
East Germany	40	50	67
West Germany	60	50	33
STIKO high-risk group, %	24	45	100
Age ≥ 60 years	–	–	100
Age ≥ 18 years + comorbidity	24	45	68
Care-facility resident	0	0	7

COVID-19, coronavirus disease 2019; IQR, interquartile range; SD, standard deviation; STIKO, Standing Committee on Vaccination.

**Table 3:** Baseline comorbidities as STIKO risk factors for severe COVID-19.

Baseline Comorbidity ^a^, %	All Recipients (n = 597)	Primary Series (n = 349)	Booster (n = 248)
≥1 STIKO high-risk comorbidity	53	45	65
≥2 STIKO high-risk comorbidity	30	23	40
≥3 STIKO high-risk comorbidity	17	13	23
Chronic neurological disease	36	29	47
Chronic intestinal disease	21	19	24
Chronic respiratory disease	11	9	13
Chronic cardiovascular disease	11	6	17
Metabolic disease (including obesity and diabetes mellitus)	10	9	10
Diabetes	6	6	4
Psychiatric disorder	5	6	4
Cancer	5	2	10
Chronic kidney disease	4	2	5
Dementia or intellectual disability	2	NA	NA
Chronic liver disease (including cirrhosis)	1	NA	NA
Autoimmune disease (including rheumatologic diseases)	NA	NA	NA
Congenital/acquired immunodeficiency or immunosuppression ^b^	NA	NA	–

NA, not available; STIKO, Standing Committee on Vaccination. ^a^ No recipients were identified as having Down syndrome. ^b^ Includes HIV infection and immunocompromised conditions post-organ transplantation due to immunosuppressive agents.

## Data Availability

Restrictions apply to the availability of these data. Data were obtained from IQVIA, and requests to the corresponding author (L.K.) will be considered with the permission of IQVIA.

## Supplementary Materials

The following supporting information can be downloaded at https://www.mdpi.com/article/10.3390/vaccines12040387/s1: Table S1. Underlying conditions in recipients at increased risk of severe COVID-19.
