# Supplementary material for: Characteristics and Outcomes for Recipients of NVX-CoV2373: A Real-World Retrospective Study in Germany
Source: Vaccines (Basel). 2024 Apr 6;12(4):387. doi: 10.3390/vaccines12040387 (PMC11054037; doi:10.3390/vaccines12040387)
Supplement: Supplementary file 1 [file vaccines-12-00387-s001.zip › vaccines-2855563-supplementary.pdf]

vaccines

SUPPLEMENT

Table S1. Underlying STIKO conditions in recipients at increased risk of severe COVID-19.

STIKO high-risk comorbidities	ICD codes	Measurement period
Congenital/acquired immunodeficiency or immunosuppression[a]	Z94, D84	3 years prior and including index
Autoimmune diseases (including rheumatological diseases)	M32 - M35, M30.3, M94.1	3 years prior and including index
Chronic cardiovascular disease	I50, I48, I20-I25	3 years prior and including index
Chronic respiratory disease	J84, J42-J46	3 years prior and including index
Chronic liver disease (including cirrhosis)	K70-K75	3 years prior and including index
Chronic kidney disease	N18, N19	3 years prior and including index
Chronic intestinal disease	K50-K52	3 years prior and including index
Chronic neurological disease[a]	G11-G13, G21-G25, G31, G36-G37, G40, G41	3 years prior and including index
Dementia or intellectual disability	F01, F03, G30, F70 – F73, F78 - F79	3 years prior and including index
Diabetes	E10-E13	3 years prior and including index
Psychiatric disorder	F43, F40 – F41, F32 – F33	1 year prior and including index
Metabolic disease (including obesity and diabetes mellitus)	E10 - E14, E66	3 years prior and including index
Down syndrome	Q90	Any time prior and including index
Cancers	C00 – C96, D00 – D49	3 years prior and including index

COVID-19, Coronavirus disease 2019; ICD, International Classification of Diseases; STIKO, Standing Committee on Vaccination.

[a]Includes chronic neuromuscular diseases.
